# Penta-Graphene as a Potential Gas Sensor for NO_*x*_ Detection

**DOI:** 10.1186/s11671-019-3142-4

**Published:** 2019-09-06

**Authors:** Meng-Qi Cheng, Qing Chen, Ke Yang, Wei-Qing Huang, Wang-Yu Hu, Gui-Fang Huang

**Affiliations:** 1 Department of Applied Physics, School of Physics and Electronics, Hunan University, Changsha, 410082 China; 2 School of Materials Science and Engineering, Hunan University, Changsha, 410082 China

**Keywords:** Penta-graphene, Adsorption, Gas sensors, First-principles calculations

## Abstract

Two-dimensional (2D) penta-graphene (PG) with unique properties that can even outperform graphene is attracting extensive attention because of its promising application in nanoelectronics. Herein, we investigate the electronic and transport properties of monolayer PG with typical small gas molecules, such as CO, CO_2_, NH_3_, NO and NO_2_, to explore the sensing capabilities of this monolayer by using first-principles and non-equilibrium Green’s function (NEGF) calculations. The optimal position and mode of adsorbed molecules are determined, and the important role of charge transfer in adsorption stability and the influence of chemical bond formation on the electronic structure of the adsorption system are explored. It is demonstrated that monolayer PG is most preferred for the NO_*x*_ (*x* = 1, 2) molecules with suitable adsorption strength and apparent charge transfer. Moreover, the current−voltage (I−V) curves of PG display a tremendous reduction of 88% (90%) in current after NO_2_ (NO) adsorption. The superior sensing performance of PG rivals or even surpasses that of other 2D materials such as graphene and phosphorene. Such ultrahigh sensitivity and selectivity to nitrogen oxides make PG a superior gas sensor that promises wide-ranging applications.

**Electronic supplementary material:**

The online version of this article (10.1186/s11671-019-3142-4) contains supplementary material, which is available to authorized users.

## Introduction

Two-dimensional (2D) materials consisting of single- or few-layer planar crystals [1], such as graphene and phosphorene, are emerging as a new paradigm in the physics of materials and have attracted increasing attention because of their unique structures and physicochemical properties [2–5], which are related to large specific surface area and fully exposed active site [6–8]. These properties endow 2D materials with very exciting prospects for wide potential applications in the fields of nanoelectronics, sensors, catalysis and solar energy conversion devices [9–16].

Penta-graphene (PG), a new 2D allotrope of carbon based on Cairo pentagonal tiling pattern, is a material with individual atomic layer exclusively consisting of pentagons (a mixture of sp^2^- and sp^3^-coordinated carbon atoms) in a planar sheet geometry [17]. Unlike graphene with zero bandgap, which greatly hinders its applications, PG has a quasi-direct intrinsic band gap of ∼ 3.25 eV, which can be tuned by doping [18, 19], hydrogenation [19] and electric field [20]. Because of its unusual atomic structure, PG has significant energetic, dynamic, thermal and mechanical stabilities up to 1000 K [17, 21, 22]. Thanks to its naturally existing bandgap and robust stability, PG may offer highly desirable properties and great potential for nanoelectronics, sensors and catalysis [23–25]. One example is that a PG-based all-carbon heterostructure shows the tunable Schottky barrier by electrostatic gating or nitrogen doping [26], verifying its potential application in nanoelectronics. Interestingly, the energy barrier of the Eley–Rideal mechanism for low-temperature CO oxidation on PG is only − 0.65 eV [25] (even comparable to many noble metal catalysts), which can be reduced to − 0.11 and − 0.35 eV by doping B and B/N, respectively [24], hence convincingly demonstrating that PG is a potential metal-free and low-cost catalyst. Recent studies also found that PG nanosheets show highly selective adsorption of NO [27], and doping can improve the adsorption of gas molecules, such as H_2_ [18], CO and CO_2_ [28] on PG. The adsorption ability of gas molecules, like graphene with good sensor properties demonstrated by both theoretical and experimental investigations [29, 30], indicates that PG would have gas-sensing properties because its electrical resistivity will be influenced by the gas molecule adsorption. However, to our best knowledge, there have been no previous reports focused on the effect of molecule adsorption on the electronic properties of PG, and given the distinctive electronic properties of PG, it is highly desirable to explore the possibility of a PG-based gas sensor.

Herein, the potential of PG monolayer as the gas sensor has been explored using density functional theory (DFT) and non-equilibrium Green’s function (NEGF) calculations. We first investigate the adsorption behaviours of several typical molecules CO_*x*_ (*x* = 1, 2), NH_3_ and NO_*x*_ (*x* = 1, 2) on PG. The preferred adsorption of NO_*x*_ on PG monolayer with the appropriate adsorption strength indicates the high selectivity of PG toward gaseous NO_*x*_. The dramatic variation in current−voltage (I−V) relation before and after NO_2_ adsorption suggests the excellent sensitivity of PG. Both the sensitivity and selectivity for gas molecules make PG a promising candidate for high-performance sensing applications.

## Methods

We perform structural relaxation and electronic calculations using first-principle calculations based on the DFT as implemented in the Vienna Ab initio Simulation Package (VASP) [31, 32]. The exchange-correlation interaction is treated within the generalized-gradient approximation (GGA) of Perdew–Burke–Ernzerhof (PBE) functional [33]. The PG model is periodic in the *xy* plane and separated by at least 15 Å along the *z*-direction. The energy cutoff is set to 450 eV and a 9 × 9 × 1 Monkhorst−Pack grid (9 × 3 × 9 for TRANSIESTA) is used for Brillouin zone integration for a 3 × 3 supercell. In order to get more accurate adsorption energy, DFT-D2 method is used. The force convergence criterion is less than 0.03 eV/Å. Spin polarization is included in the calculations of the adsorption of NO_*x*_ because they are paramagnetic. The transport properties are studied by the non-equilibrium Green’s function (NEGF) method as implemented in the TRANSIESTA package [34]. The electric current through the contact region is calculated using the Landauer−Buttiker formula [35], $I\left({V}_b\right)={G}_0\;{\int}_{\mu_L}^{\mu_R}T\;\left(E,{V}_b\right) dE$, where *G*_0_ and *T* are the quantum conductance unit and the transmission rate of electrons incident at energy *E* under a potential bias *V*_*b*_, respectively. The electrochemical potential difference between the two electrodes is *eV*_*b*_ = *μ*_*L*_ − *μ*_*R*_.

## Results and Discussion

**Fig. 1:**
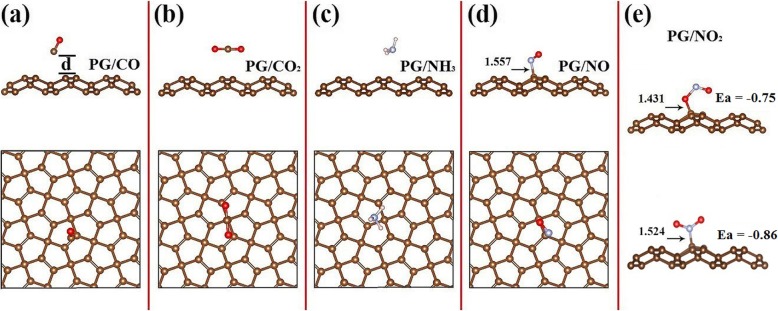
Adsorption configurations. **a**–**d** Side view (top) and top view (bottom) of the fully relaxed structural models of penta-graphene (PG) with CO, CO_2_, NH_3_ and NO adsorption, respectively. The last one (**e**) is the side view of the two bonding modes when NO_2_ is adsorbed, the binding energy (Ea) has been given. The distance between the gas molecule and the penta-graphene layer is indicated in **a** and the bond lengths between C and N (**d**, **e**) and C and O (**e**) at the interface are given (in angstrom units). For simplicity, these structural models are abbreviated as **a** PG/CO, **b** PG/CO_2_, **c** PG/NH_3_, **d** PG/NO and **e** PG/NO_2_

Before investigating the structural characteristics and energetics of an adsorption system, we first optimize the lattice constants of the monolayer PG and obtain *a* = *b* = 3.63 Å, in agreement with previous reported values [17]. To find the most favourable configurations, different adsorption sites and orientations are examined to adsorb gas molecules, each of them being placed on a 3 × 3 supercell PG. After full relaxation, we find that the NO_*x*_ molecules chemically adsorb on PG via strong chemical bonds, whereas the other three molecules (CO_*x*_, NH_3_) are physically adsorbed (Fig. 1). The CO, CO_2_ and NH_3_ molecules are staying above PG with an adsorption distance of 2.40, 2.73 and 2.43 Å, respectively (Table 1), showing a weak van der Waals interaction between them. By contrast, the dipolar NO_*x*_ molecule is attracted to the top position of a C atom, forming a chemical bond with bond length to be 1.43~1.56 Å. Note that for PG/NO_2_, both N and O atoms can chemically be bonded to C atom in PG (Fig. 1e).

**Table 1:** The bandgap *E*_gap_ (eV), interfacial spacing (*d*), and bader charge analysis of optimized PG with gas molecules

Structure	*E*_gap_ (eV)	*d* (Å)	Bader charge (e)	
			Molecules	PG
PG/CO	2.35	2.40	0.006	− 0.006
PG/CO_2_	2.37	2.73	0.023	− 0.023
PG/NH_3_	2.34	2.43	− 0.011	0.011
PG/NO	1.14	1.52	0.243	− 0.243
PG/NO_2_	1.62	1.29	0.517	− 0.517

The stability of molecules on PG is evaluated by the adsorption energy (*E*_*a*_), defined as *E*_*a*_ *= E*_pg *+* gas_ *− E*_gas_ *− E*_pg_ where *E*_pg *+* gas_, *E*_pg_ and *E*_gas_ are the total energies of gas-absorbed PG, pristine PG and isolated molecule, respectively. Table 2 shows that similar to graphene and phosphorene in their potential use as gas sensors [29, 36], the adsorption energies of PG/NO and PG/NO_2_ are *−* 0.44 eV and *−* 0.75 eV per molecule, respectively (approaching *−* 0.5 eV, which is taken as the reference for gas capture), which are big enough to withstand the thermal disturbance at room temperature that is at the energy scale of *k*_B_*T* (*k*_B_ is the Boltzmann constant) [36]. However, the adsorption energies of PG/CO_*x*_ and PG/NH_3_ are small (*−* 0.05 to approximately *−* 0.11 eV), indicating that the CO_*x*_ and NH_3_ molecules cannot readily be adsorbed on PG. The results verify that monolayer PG has a high selectivity to toxic NO_*x*_ gas. More importantly, the sensing characteristics of PG for NO_*x*_ is unique compared to other 2D nanosheets, such as graphene, silicene, germanene, phosphorene and MoS_2_, which they fail to distinguish NO_*x*_ and/or CO_*x*_ (NH_3_), as shown in Table 2.

**Table 2:** Comparative summary of adsorption of molecules (adsorption energies in eV) on different 2D surfaces collected from literature vs on the penta-graphene surface

Surface	Molecules				
	CO	CO_2_	NH_3_	NO	NO_2_
Penta-graphene	− 0.05	− 0.10	− 0.11	− 0.44	− 0.75
Graphene [30, 37]	− 0.01	− 0.05	− 0.03	− 0.03	− 0.07
Silicene [38]	− 0.18	− 0.04	− 0.60	− 0.35	− 1.37
Germanene [39, 40]	− 0.16	− 0.10	− 0.44	− 0.51	− 1.08
Phosphorene [36]	− 0.32	− 0.41	− 0.50	− 0.86	− 0.60
MoS_2_ [41, 42]	− 0.44	− 0.33	− 0.16	− 0.55	− 0.14

**Fig. 2:**
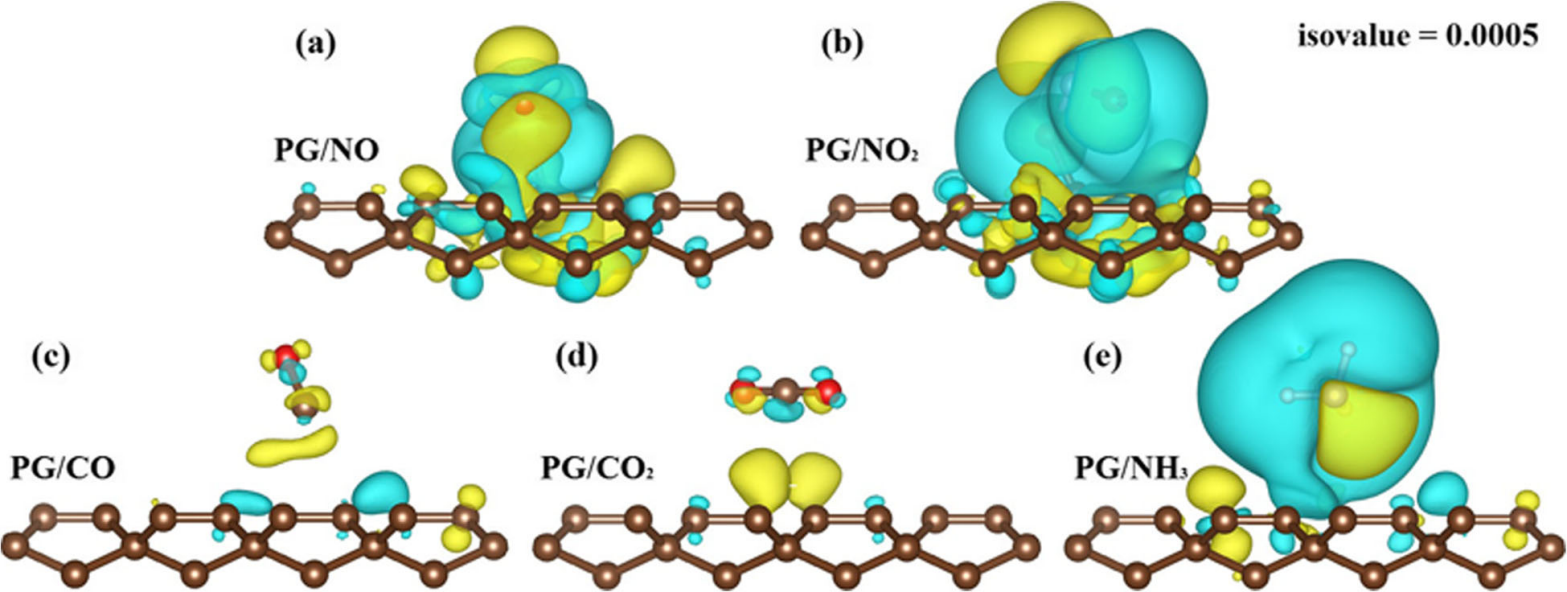
Charge density difference plots. The adsorption configurations and charge transfer for each case in a different order from Fig. 1 are plotted in **a**–**e**. The yellow isosurface indicates an electron gain, while the blue one represents an electron loss. The unit of isosurface value is *e* Å^−3^. Apparently, electron transfer in covalent **a** PG/NO and **b** PG/NO_2_ structure are much more obvious than others

It has been demonstrated that in most cases, gas adsorption plays an important role of charge transfer in determining the adsorption energy and causing a change in the resistance of the host layer. We first calculate the interfacial charge transfer, which can be visualized in a very intuitive way, by the 3D charge density difference, *Δρ* = *ρ*_tot_ (*r*) − *ρ*_pg_(*r*) − *ρ*_gas_(*r*), where *ρ*_tot_(*r*),*ρ*_pg_(*r*) and *ρ*_gas_(*r*) are the charge densities of PG with and without gas adsorption and free gas molecule in the same configuration, respectively [43]. Figure 2 shows the calculated electron transfer for the adsorption of NO_*x*_, CO_*x*_ and NH_3_ on PG, respectively. Obviously, the charge density variation is significant at the interface. Compared to the chemically adsorbed NO_*x*_ systems, the charge redistribution at the PG/CO and PG/CO_2_ interfaces is relatively weak. This is due to the stronger interaction between covalent bonds than van der Waals forces. As for NH_3_ adsorption on PG, the charge redistribution occurs around the NH_3_ molecule.

A further charge analysis based on the Bader method can give a more quantitative measure of charge redistribution in these systems, which are listed in Table 1. As expected, for the physical adsorption of CO_*x*_ and NH_3_ on PG, only a small amount (< 0.025 e) of charge is transferred between PG and gas molecules, further illuminating a weak binding. By contrast, the amount of charge transfer in the chemically adsorbed systems is more than 10 times higher: up to 0.517 e (0.243 e) is transferred from the PG layer to the NO_2_ (NO) molecule (Table 1), in agreement with their stronger adsorption energy. This systematic trend in adsorption strength correlated with the charge transfer helps us to understand the mechanism for gas molecule adsorption on PG and also indicates that the gas adsorption can be controlled by an electric field, similar to the case of gas NO_*x*_ (*x* = 1, 2) molecules absorbed on monolayer MoS_2_ [9].

**Fig. 3:**
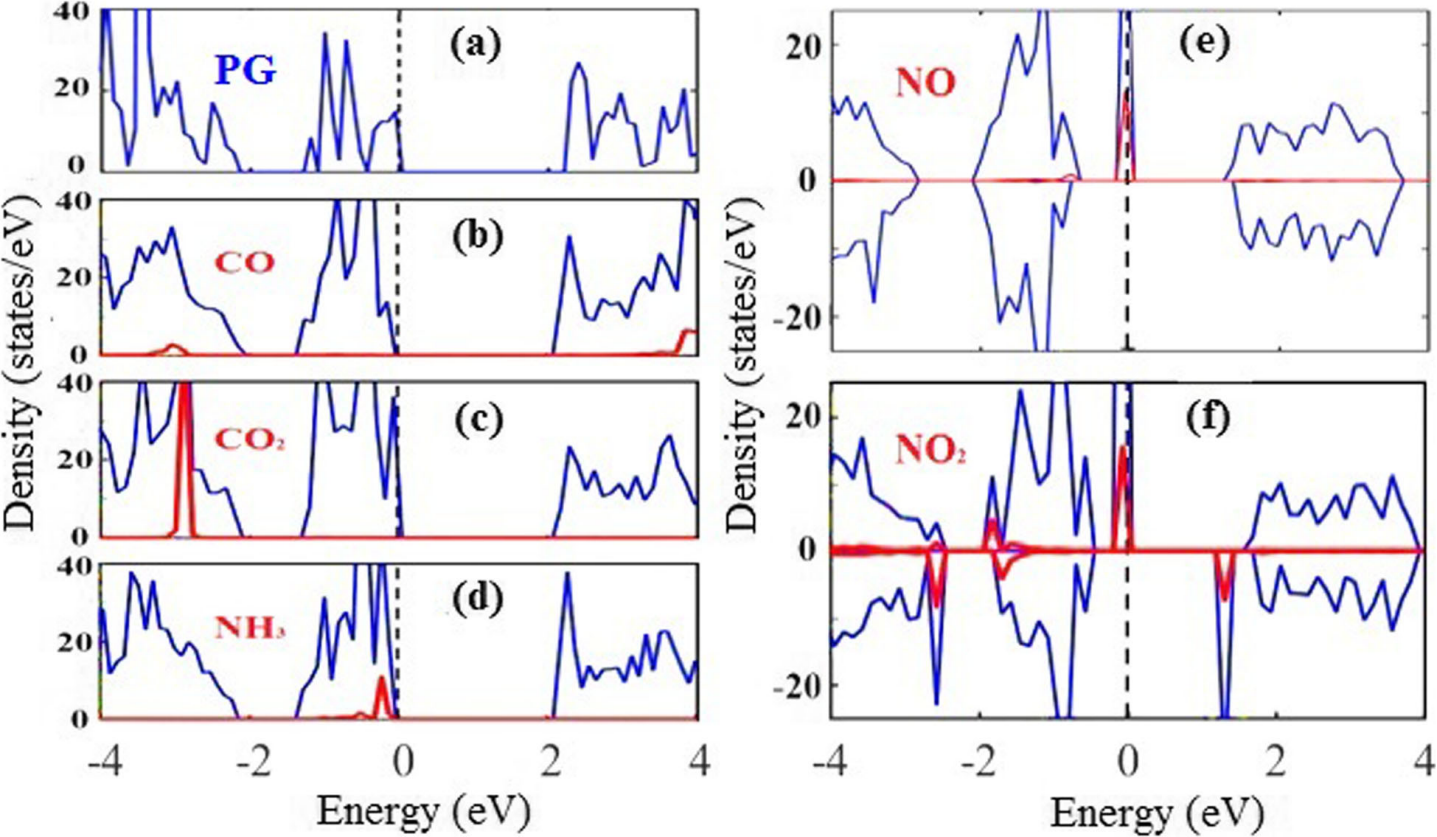
Total electronic density of states. **a** The DOS of pristine penta-graphene. **b**–**f** Total DOS of penta-graphene with each gas molecule adsorption (blue lines) and the partial DOS from the gas molecule (red lines). The Fermi level is taken to be zero and displayed with a black dashed line

We next investigate the effects of gas adsorption on the electronic properties of PG. Figure 3 displays the total density of states (DOS) of PG without and with the gas molecule adsorption, as well as the projected DOS from corresponding individuals. A bandgap of 2.10 eV is obtained, in agreement with previous DFT results of pure PG [44], due to the fact that the PBE/GGA functional usually underestimate the band gap of semiconductors. Although this will affect the threshold bias (i.e. the voltage that can produce observable current), it is expected to not affect other transport properties, as will be demonstrated in the following. Figure 3a shows the DOS of pristine PG and Fig. 3b and c shows that the DOS near the valence band (VB) or conduction band (CB) of PG is not obviously affected by the CO_*x*_ adsorption, in perfect line with their small adsorption energies and the weak charge redistribution. Although the adsorption of NH_3_ molecule leads to a small state near the VB top (Fig. 3d), the physical adsorptions of molecules do not alter noticeable variations of the DOS near the Fermi level. These results indicate that the adsorption of CO_*x*_ and NH_3_ does not have a significant effect on the electronic structure of PG. By striking contrast, distinct hybridizing states are observable near the Fermi level for NO_*x*_-adsorbed PG sheet, as plotted in Fig. 3e and f. This feature, combining with major charge density redistributions, demonstrates a stronger interaction between NO_*x*_ and PG monolayer, resulting into appreciable band structure modifications. This will have a great impact on the transport properties of PG, making it a very sensitive gas sensor.

Studies have demonstrated that some 2D materials are extremely sensitive to gas molecule adsorption, which corresponds to extremely low densities of gas molecules. In order to simulate the gas concentration-dependent sensitivity of PG, we calculated the effect of coverage of adsorbed gas on the properties of PG. Take the PG/NO system as an example, when the coverage is 5.56%, the adsorption energy is about − 0.44 eV per molecule. As the coverage decreases to 3.13~2.0%, the adsorption energy is reduced to about − 0.32 eV per molecule. This indicates that the gas concentration variation does not change the main conclusions. In the following calculations, therefore, the PG/NO system model with 5.56% coverage (using the 3 × 3 supercell) is chosen as a representative to calculate electronic and transport properties.

**Fig. 4:**
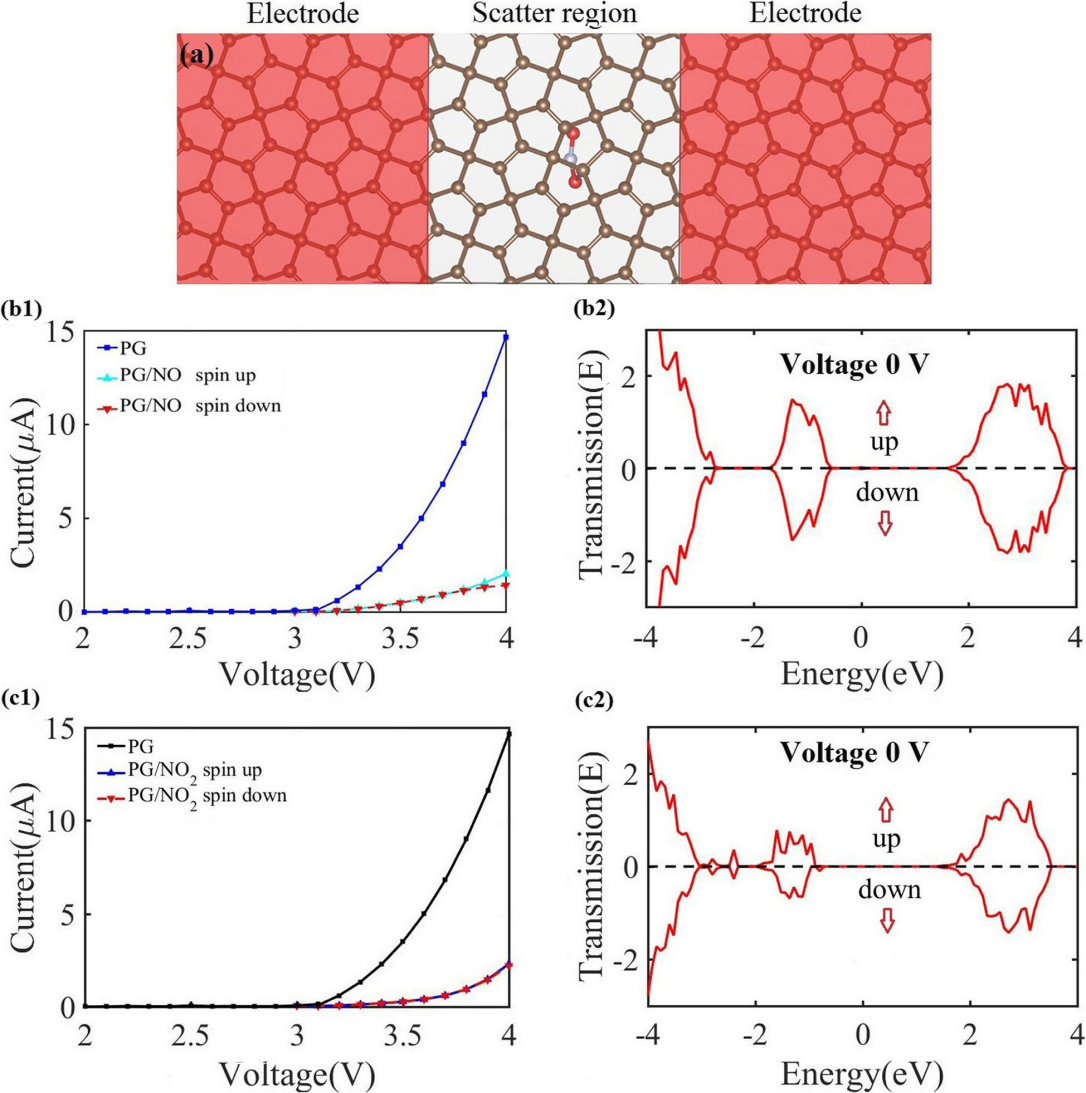
Illustration of the two-probe systems (**a**) where semi-infinite left and right electrode regions (red shaded region) are in contact with the central scattering region. For the electrodes and scatter regions, 3 × 3 supercells without and with NO are used, respectively. In **b1** and **c1**, we display the I−V curves of pure PG and PG with the NO and NO_2_ adsorption. The transmission spectra under zero bias are shown in **c1** and **c2**

To qualitatively evaluate the sensitivity of PG monolayer for NO_*x*_ monitoring, we employ the NEGF method to simulate the transport transmission and current–voltage (I–V) relations before and after the NO_*x*_ adsorption using the two-probe models, as plotted in Fig. 4 a. To make the physical picture clearer and also reduce the burden of calculations, a two-probe system (pseudo “device” structure) is used, in which the “fake electrodes” just built from the periodic extension of the clean nanosheet, just as widely used in previous works [36]. Here, a 3 × 3 PG supercell (the same as the electronic calculations) without and with gas adsorption is used for each of the left and right electrodes, and the center scattering region, respectively (Fig. 4a). For comparison, the same calculations for the center scattering region without gas adsorption is performed. The calculated I−V curves of PG with and without the NO_*x*_ adsorption are shown in Fig. 4b1 and 4c1. The adsorption of paramagnetic molecule NO_*x*_ on PG induces spin polarization, thus leading to spin-polarized current. When a bias voltage is applied, the Fermi level of the left shifts upward with respect to that of the right electrode. Therefore, the current starts to flow only after the VB maximum of the left electrode reaches the CB minimum of the right electrode [36]. As a result, there is no current passing through the center scattering region when the bias voltage is smaller than 3.25 V, which is close to the intrinsic gap of PG [17]. When the bias voltage increases from 3.25 V, the currents in both spin channels increase rapidly. Under a bias of 3.9 V, the current passing through the PG without gas adsorption is 13.4 μA; however, as PG absorbs NO_2_ molecule, the current under the same bias is sharply decreased to 1.6 μA, which is about an 88% reduction. Moreover, when PG absorbs NO molecule, the current is decreased to 1.34 μA, which is about a 90% reduction. To explore the coverage effect, we further consider one molecule adsorbed on the 4 × 4 and 5 × 5, as displayed in Additional file 1: Figure S1. One can see that the interaction between molecules and the PG sheet does not change much with coverage, resulting in similar adsorption energy *E*_*a*_. The transport properties of PG/NO with a 5 × 5 supercell central region is calculated and given in Additional file 1: Figure S2. Under the bias of 3.9 V, the current through a 5 × 5 supercell central region with one NO molecule is decreased to 2.87 μA (about a 79% reduction). The dramatic reduction of current indicates a significant increase of resistance after the NO_*x*_ adsorption, which could be directly measured in experiment. The significant change in current signifies the ultrahigh sensitivity of the PG sensor to NO_*x*_, which rivals or even surpasses that of other 2D nanosheets such as silicene and phosphorene [36, 38], as clearly displayed in Table 2.

To elucidate the mechanism of the increased resistance of NO_*x*_-adsorbed PG, the transmission spectra of PG with NO_2_ adsorption under zero bias are calculated and displayed in Fig. 4 c. One can see that a region of zero transmission with a width of 3.25 V is observed around the Fermi level, and beyond this region, there are mountain-like characteristics in the transmission spectra. The same trend of DOS (Fig. 3f) proves that the choice of PBE functional does not have a huge impact on the electronic structure and transport properties. Figure 3f shows that the lowest unoccupied molecular orbital (LUMO) state and highest occupied molecular orbital (HOMO) state are located at the gap edge, which is mainly formed by the *p*_*z*_ orbitals. As the charge transfers from the C *p*_*z*_ orbitals to the NO_2_ molecule, the LUMO and HOMO states can be obviously affected by NO_2_ adsorption. This indicates that the adsorbed NO_2_ molecule becomes strong scattering centers for charge carriers, thus resulting into a degraded mobility due to the local state around the zone center induced by NO_2_ molecule. In other words, the obstructed conducting channels lead to a shorter carrier lifetime or mean free path and thus a smaller mobility in NO_*x*_-adsorbed PG.

As one of the important factors for gas sensor, the recovery time is worthy to consider, which is the time taken by the sensor to get back 80% of the original resistance. According to the transition state theory [45], the recovery time τ can be calculated by the formula*τ* = *ω*^‐1^ exp(*E*^∗^/*K*_B_*T*), where *ω* is attempt frequency (~10^13^ s^−1^ according to previous report [46, 47]), *T* is temperature and *K*_B_ is Boltzmann constant (8.318 × 10^-3^ kJ/(mol*K)), the *K*_B_*T*is about 0.026 eV at room temperature, E* is the desorption energy barrier. One can see that the recovery time is closely related to the desorption barrier: the lower the desorption barrier, the shorter the recovery time of NO_*x*_ on PG surface at the same temperature. Given that desorption could be considered as the inverse process of adsorption, it is reasonable to assume that the value of *E*_ad_ to be the potential barrier (E^∗^). Thus, the potential barriers (E^∗^) for PG/NO and PG/NO_2_ are 0.44 eV and 0.75 eV, respectively. The calculated response times of the two systems are respectively 2.24 × 10^−6^ s and 0.34 s at the temperature of 300 K, indicating that the PG sensor is able to completely recover to its initial states. From the results given above, one can conclude that the PG is a potential material for NO_*x*_ gas with a high sensitivity and quick recovery time.

## Conclusions

In this work, we have systematically investigated the structural, electronic, and transport properties of the PG monolayer with the adsorption of typical gas molecules using DFT calculations. The results show that PG monolayer is one of the most preferred monolayer for toxic NO_*x*_ gases with suitable adsorption strength compared to other 2D materials such as silicene and phosphorene. The electronic resistance of PG displays a dramatic increase with the adsorption of NO_2_, thereby signifying its ultrahigh sensitivity. In a word, PG has superior sensing performance for NO_*x*_ gas with a high sensitivity and quick recovery time. Such unique features manifest the monolayer PG a desirable candidate as a superior gas sensor.

## Additional File

Additional file 1: **Figure S1** (a-c) Side (top) and top (bottom) views of the fully relaxed structural PG/NO models of different supercells (from 3×3 to 5×5). The binding energy (*E*a) is denoted. **Figure S2** (a) Illustration of the two-probe systems where semi-infinite left and right electrode regions (red shade region) are in contact with the central scattering region. For the electrodes and scatter regions, 3 × 3 supercells without NO and 5 × 3 supercells with NO are used, respectively. In (b) we display the I−V curves of pure PG and PG with the NO adsorption. (DOCX 281 kb)

## Data Availability

The datasets supporting the conclusions of this article are included within the article, and further information about the data and materials could be made available to the interested party under a motivated request addressed to the corresponding author.

## Abbreviations

2D: Two-dimensional
CB: Conduction band
DFT: Density functional theory
GGA: Generalized-gradient approximation
HOMO: Highest occupied molecular orbital
LUMO: Lowest unoccupied molecular orbital
NEGF: Non-equilibrium Green’s function
PBE: Perdew–Burke–Ernzerhof
PG: Penta-graphene
PG/CO: Penta-graphene with CO adsorption
PG/CO_2_: Penta-graphene with CO_2_ adsorption
PG/NH_3_: Penta-graphene with NH_3_ adsorption
PG/NO: Penta-graphene with NO adsorption
PG/NO_2_: Penta-graphene with NO_2_ adsorption
VASP: Vienna Ab initio Simulation Package
VB: Valence band
