# Supplementary material for: Penta-Graphene as a Potential Gas Sensor for NOx Detection
Source: Nanoscale Res Lett. 2019 Sep 6;14:306. doi: 10.1186/s11671-019-3142-4 (PMC6730973; doi:10.1186/s11671-019-3142-4)
Supplement: Supplementary file 1 — Figure S1 (a-c) Side (top) and top (bottom) views of the fully relaxed structural PG/NO models of different supercells (from 3×3 to 5×5). The binding energy (Ea) is denoted. Figure S2 (a) Illustration of the two-probe systems where semi-infinite left and right electrode regions (red shade region) are in contact with the central scattering region. For the electrodes and scatter regions, 3 × 3 supercells without NO and 5 × 3 supercells with NO are used, respectively. In (b) we display the I−V curves of pure PG and PG with the NO adsorption. (DOCX 281 kb) [file 11671_2019_3142_MOESM1_ESM.docx]

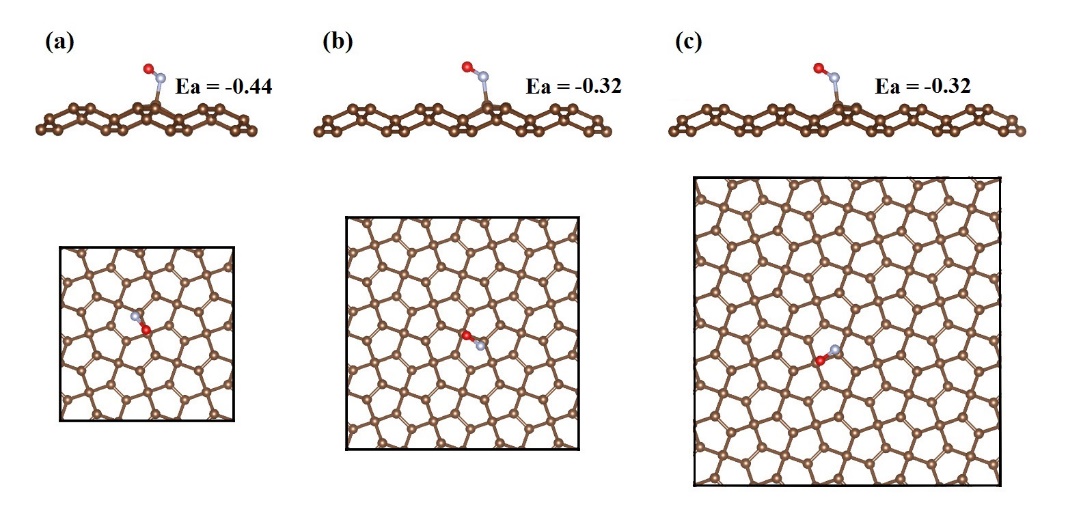


**Figure S1** (a-c) Side (top) and top (bottom) views of the fully relaxed structural PG/NO models of different supercells (from 3×3 to 5×5). The binding energy (*E*a) is denoted.


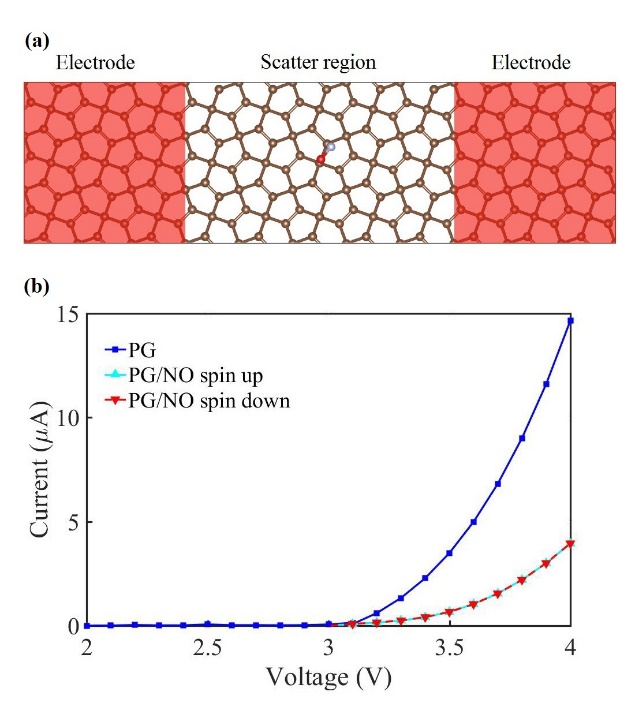


**Figure S2** (a) Illustration of the two-probe systems where semi-infinite left and right electrode regions (red shade region) are in contact with the central scattering region. For the electrodes and scatter regions, 3 × 3 supercells without NO and 5 × 5 supercells with NO are used, respectively. In (b) we display the I−V curves of pure PG and PG with the NO adsorption.
